# A triple-layer, autograft-free reconstruction strategy for skull base repair after standard endoscopic endonasal resection of pituitary adenomas

**DOI:** 10.3389/fsurg.2026.1851438

**Published:** 2026-07-13

**Authors:** Wenxuan Xin, Maosheng Xiang, Junjie Liu, Zijia Guo, Nan Bai, Junhao Zhu, Yuanming Geng, Weiyi Xie, Zixiang Cong, Chiyuan Ma

**Affiliations:** 1 Jinling Clinical Medical College, Nanjing University of Chinese Medicine, Nanjing, China; 2 Department of Neurosurgery, Nanjing Jinling Hospital, Affiliated Hospital of Medical School, Nanjing University, Nanjing, China; 3 Jinling Clinical Medical College, Nanjing Medical University, Nanjing, China

**Keywords:** cerebrospinal fluid rhinorrhea, dura mater, endoscopy, pituitary adenoma, skull base

## Abstract

**Objective:**

It is imperative to prevent cerebrospinal fluid (CSF) leakage following an endoscopic endonasal approach (EEA). This study aims to evaluate the efficacy of a simple, triple-layer reconstruction strategy for preventing CSF rhinorrhea following the standard EEA for pituitary adenomas.

**Methods:**

We retrospectively analyzed 213 patients who underwent a standard EEA for primary pituitary adenomas between January 2023 and March 2025. All patients received a simple reconstruction consisting of artificial dura mater placement, gelatin sponge packing, and porcine fibrin sealant sealing, without autograft harvesting. Intraoperative CSF leakage was graded according to the Kelly classification. Postoperative outcomes including CSF rhinorrhea, meningitis, and subjective olfactory changes were recorded during at least three months of follow-up. The study focused exclusively on reconstruction-related outcomes and did not assess other endpoints such as gross total resection, endocrine remission, visual recovery, and diabetes insipidus. This retrospective, single-arm case series reports preliminary outcomes of a triple-layer reconstruction strategy.

**Results:**

Intraoperative CSF leakage occurred in 97 patients (45.54%): Grade 1 in 41, Grade 2 in 45, and Grade 3 in 11. Postoperative CSF rhinorrhea developed in 3 of 213 patients (1.41%; 95% CI: 0.29%–4.25%). Among the 97 patients with intraoperative CSF leakage, the postoperative leak rate was 3.1% (3/97; 95% CI: 0.67%–9.08%). Among the 11 patients with Grade 3 intraoperative leaks, no postoperative CSF rhinorrhea was observed in this series. Meningitis occurred in 4 patients (1.88%), two of whom also had CSF rhinorrhea. Postoperative hyposmia was reported by 2 patients (0.94%), both of whom recovered normal olfaction by 6 months.

**Conclusions:**

In this retrospective case series, the triple-layer reconstruction strategy was associated with a low postoperative CSF leak rate following standard EEA for primary pituitary adenomas. Among the small number of patients with Grade 3 intraoperative leaks in this series, no postoperative CSF rhinorrhea was observed. The technique also avoided autograft harvest and donor-site morbidity. These findings should be considered preliminary and require confirmation in prospective, controlled studies.

## 1 Introduction

The standard EEA (1) remains the preferred surgical method for most pituitary lesions (2–4) CSF rhinorrhea continues to be one of the most challenging complications following EEA, and its effective management directly impacts patient outcomes. Current skull base reconstruction strategies have established standardized protocols based on defect size and CSF leak grading (5). The vascularized nasoseptal flap (VNSF) combined with multi-layered reconstruction is considered the mainstream approach for skull base reconstruction (3, 6–18), but it involves multiple meticulous steps and carries donor site complications such as wound dehiscence, infection, and decreased sense of smell (19). Unlike the extensive defects created in expanded EEA, CSF leaks in standard EEA typically originate from a rupture of the sellar arachnoid rather than the suprasellar dura mater. This anatomical characteristic suggests that the management of a CSF leak following standard EEA hinges on the arachnoid layer, potentially without necessitating the complexity of a vascularized flap for every case. In response to the characteristics of CSF leak following standard EEA, we propose a simplified reconstruction approach. Herein, we report our preliminary experience with this simplified technique for skull base reconstruction.

## 2 Methods

### 2.1 Inclusion and exclusion criteria

**Figure 1:**
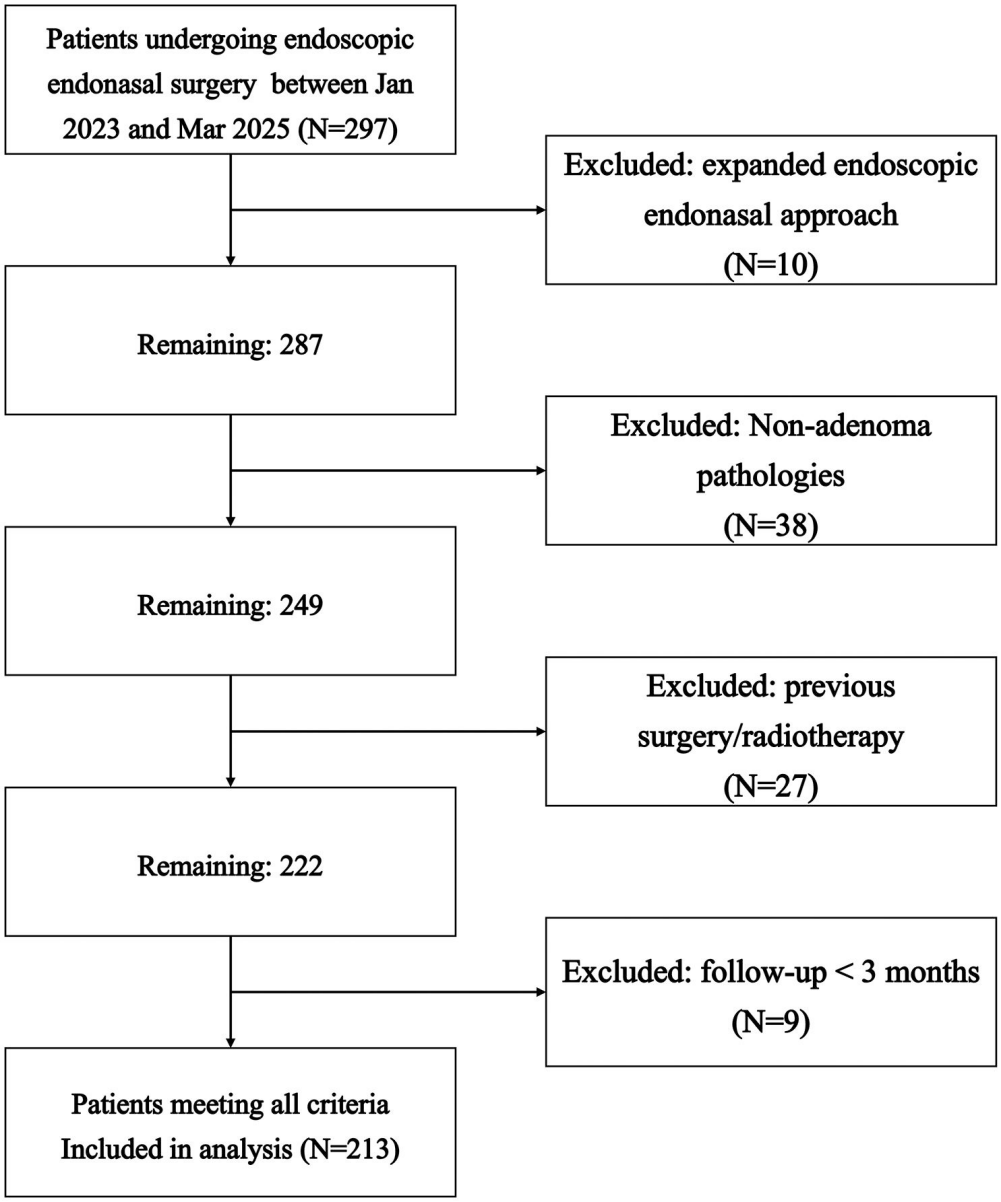
Flow chart for patient selection. A total of 297 patients undergoing endoscopic endonasal surgery between January 2023 and March 2025 were initially screened. Among these, 10 were excluded because the procedure involved an expanded endonasal approach, 38 were excluded due to non-adenoma pathologies, 27 were excluded due to prior surgery or radiotherapy, and 9 were excluded because follow-up duration was less than three months. The remaining 213 patients were included in the final analysis.

This was a retrospective, descriptive, single-center case series. Patients were eligible for inclusion if they: (1) underwent a standard EEA resection of pituitary adenoma at Jinling Hospital between January 2023 and March 2025; (2) had complete intraoperative and follow-up data available. Exclusion criteria were: (1) previous transsphenoidal surgery, transcranial surgery for pituitary, or radiotherapy to the sellar region; (2) presence of other skull base pathologies (e.g., craniopharyngioma, meningioma); (3) follow-up duration less than three months (Figure 1). During the study period, all patients who met the inclusion criteria received the triple-layer reconstruction technique consecutively. No eligible patient received alternative reconstruction methods.

### 2.2 Data collection and variables

Demographic, clinical, and operative data were extracted from electronic medical records. Collected variables included:

Baseline characteristics: age, sex, pathological subtype (nonfunctioning vs. functioning adenomas, further categorized by hormone secretion).

Tumor characteristics: Tumor size was classified based on the maximal diameter measured on preoperative MRI as microadenomas (<10 mm), macroadenomas (10–40 mm), and giant adenomas (>40 mm). Cavernous sinus invasion was assessed using the Knosp classification (grades 0-4) (20) on coronal MRI.

Operative details: intraoperative CSF leak graded using Kelly classification system (Grade 0-3) (21), operative duration.

Postoperative outcomes: occurrence of CSF rhinorrhea, meningitis, and subjective olfactory changes (hyposmia/anosmia) assessed at follow-up visits.

### 2.3 Surgical technique

All procedures were performed by the same team of experienced surgeons (Chiyuan Ma, Zixiang Cong) using endoscopic 1½-transseptal approach (22). A cross-shaped incision is made in the sella floor dura mater to fully expose the lesions within the pituitary fossa. Following tumor resection, the arachnoid membrane collapses into the sella, and the anatomical site of CSF leakage during surgery is located here.

**Figure 2:**
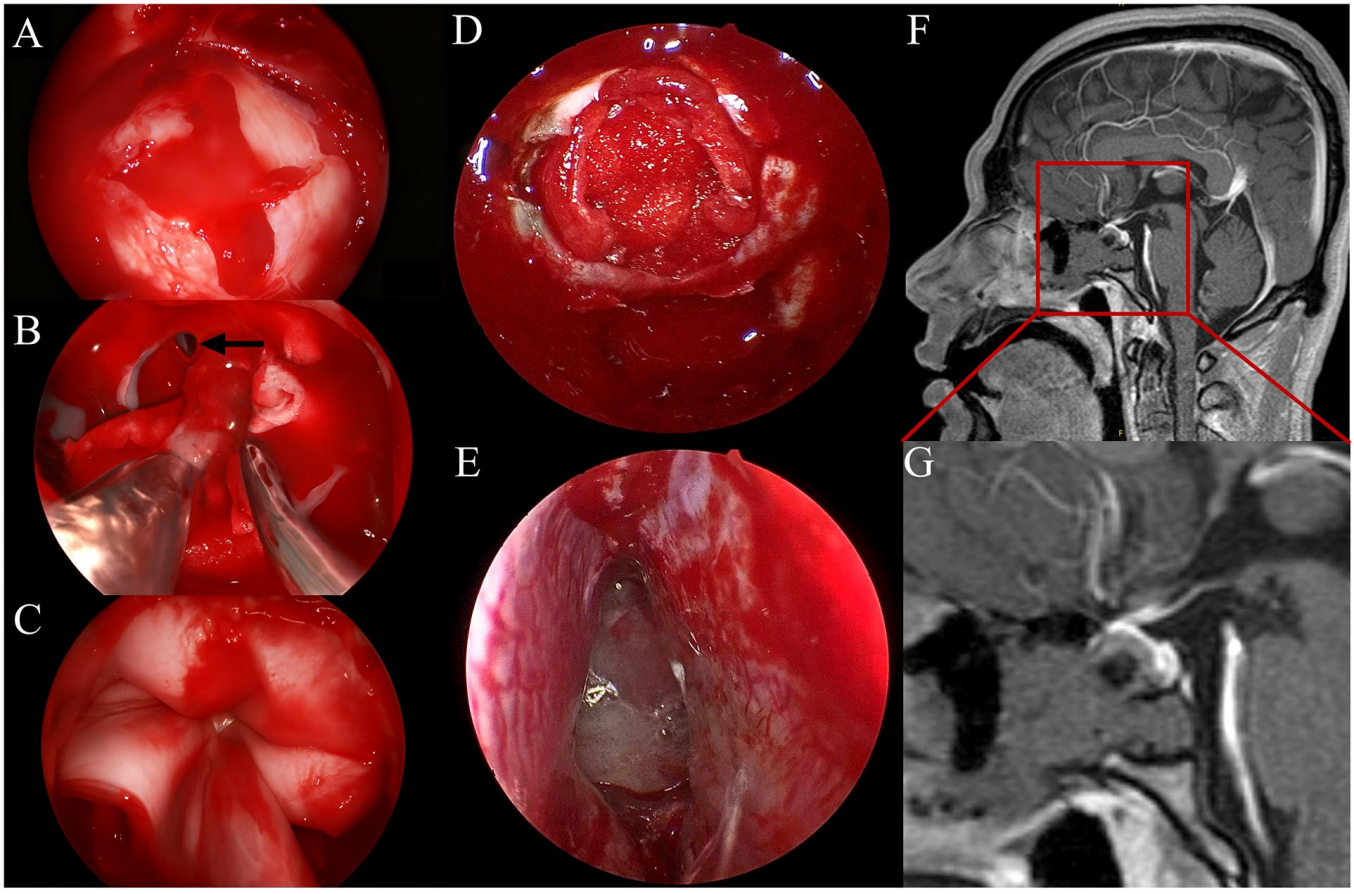
Simple reconstruction surgery technique diagram. **(A)** Make a cross-shaped incision through the dura mater at the base of the sella turcica, preserving the supporting structures at the four corners. **(B)** Following tumor resection, an arachnoid fistula (black arrow) was observed. Intraoperative CSF leakage was classified as Grade 3. **(C)** Cover the entire diaphragma sellae and the medial wall of the sellar region with artificial dura mater. Black star: Pituitary fossa. **(D)** Gelatin sponge packing of the pituitary fossa ensures full contact between artificial dura mater and the diaphragma sellae, directly covering the leak. **(E)** Porcine fibrin sealant sealing and fixation. **(F,G)** Postoperative pituitary MRI.

First, the artificial dura mater (Dural Graft Matrix, Integra LifeSciences) was trimmed to a size sufficient to cover the entire tumor cavity and placed as an inlay construct. Second, the gelatin sponges (Xiang'en Medical Technology Development Co. Ltd.) were used to fill the entire cavity, ensuring that the artificial dura adhered tightly to the arachnoid layer and lay flat over the cavity. The four preserved corners of the cruciate dural incision provided external structural support, helping to keep the packing material in place. Overpacking was strictly avoided, especially in macroadenomas where the cavity is large, as excessive packing could compress the optic nerve. The packing firmness was guided by tactile feedback, approximating the texture of a nasal tip. Finally, the surgical area was covered with neuropatties to avoid displacement of the reconstructed material, and then the liquid around the surgical area was absorbed using a suction through the neuropatties. Porcine fibrin sealant (Guangzhou Beixiu Bio-Technology Co., Ltd., Guangzhou, China; trade name: Beixiu Glue) was then injected to seal the construct and reinforce the closure. In addition, the sellar floor was not reconstructed. No rigid buttress and nasal packing were used (Figure 2). In this series, no case required conversion to a vascularized flap, autologous graft, or reoperation. Lumbar drainage (LD) was not used prophylactically but was reserved as salvage therapy for postoperative leaks or meningitis.

### 2.4 Outcome definitions and assessments

CSF rhinorrhea: defined as persistent clear nasal discharge with positive glucose test (>1/3 of serum glucose) or confirmed via imaging. *β*-2 transferrin testing was not routinely available at our institution during the study period; therefore, glucose testing was used as the standard method despite its lower specificity.

Meningitis: diagnosed based on clinical symptoms (fever, headache, neck stiffness) plus CSF analysis (leukocytosis >1000*10^6^/L, elevated protein, low glucose) and/or positive culture.

Olfactory function: assessed via patient self-report during follow-up.

### 2.5 Postoperative management protocol

Patients were transferred to the neurosurgical ward for close monitoring. Routine vital sign and neurological assessments were performed every 4 h for the first 24 h. Intravenous antibiotics were administered for 48 h. Strict bed rest with the head elevated to 30 degrees was maintained for 24–48 h. Patients were instructed to avoid bending over, straining, nose blowing, coughing, and sneezing. Ambulation was gradually resumed thereafter with continued avoidance of Valsalva maneuvers. A soft diet was initiated on postoperative day 1. Stool softeners and laxatives were routinely prescribed to prevent constipation and associated increases in intracranial pressure. Patients were typically discharged on postoperative day 7 if no complications occurred. Detailed written and verbal instructions were provided regarding activity restrictions and signs of potential complications. The first outpatient follow-up was scheduled at 4 weeks postoperatively, with subsequent visits at 3 and 6 months.

### 2.6 Statistical analysis

Statistical analysis was performed using R statistical software (version 4.4.3). Continuous variables are presented as mean ± standard deviation (SD), categorical variables as frequencies and percentages. Ninety-five percent confidence intervals (CIs) for proportions were calculated using the Clopper-Pearson (exact) method. Due to the small number of postoperative CSF rhinorrhea events (*n* = 3), formal hypothesis testing was not performed, and all comparisons are descriptive only.

## 3 Results

### 3.1 Baseline and tumor characteristics

Of the 297 patients initially screened, 10 were excluded due to expanded endoscopic endonasal approach, 38 due to non-adenoma pathologies, 27 due to prior surgery or radiotherapy, and 9 due to follow-up of less than three months (Figure 1). A total of 213 patients who underwent standard endoscopic endonasal resection of primary pituitary adenomas were included in this study. All patients had a minimum follow-up of 3 months. A total of 197 patients (92.49%) completed the 6-month follow-up; the remaining 16 patients were lost to follow-up after the 3-month visit. The case series comprised 87 males (40.85%) and 126 females (59.15%), with a mean age of 52.62 ± 12.99 years (Table 1).

**Table 1:** Baseline demographic and tumor characteristics (*n* = 213).

Characteristic	Sum
Age (mean ± SD)	52.62 ± 12.99 years
Gender	
Male	87 (40.85%）
Female	126 (59.15%)
Pathology	
Nonfunctioning pituitary adenoma	139 (65.26%）
Functioning pituitary adenoma	74 (34.74%)
GH	29 (13.62%)
PRL	33 (15.49%)
ACTH	12 (5.63%)
Tumor size category	
Microadenoma	26 (12.21%)
Macroadenoma	142 (66.67%)
Giant adenoma	45 (21.12)
Knosp grade	
0	52 (24.41%)
1	54 (25.35%)
2	54 (25.35%)
3	34 (15.96%)
4	19 (8.92%)

Regarding pathological subtypes, 139 patients (65.26%) had nonfunctioning adenomas, while 74 patients (34.74%) had functioning adenomas. Among the latter, growth hormone (GH)-secreting, prolactin (PRL)-secreting, and adrenocorticotropic hormone (ACTH)-secreting adenomas accounted for 13.62% (*n* = 29), 15.49% (*n* = 33), and 5.63% (*n* = 12) of the case series, respectively.

Based on preoperatively MRI, tumor size was categorized as follows: microadenomas (<10 mm) in 26 patients (12.2%), macroadenomas (10–40 mm) in 142 patients (66.7%), and giant adenomas (> 40 mm) in 45 patients (21.1%). Cavernous sinus invasion distribution based on Knosp grade. Grade 0 (*n* = 52, 24.4%), Grade 1 (*n* = 54, 25.4%), Grade 2 (*n* = 54, 25.4%), Grade 3 (*n* = 34, 16.0%), and Grade 4 (*n* = 19, 8.9%). The overall mean maximal tumor diameter was 23.4 ± 11.2 mm.

### 3.2 Intraoperative outcomes and operative duration

Intraoperative CSF leakage occurred in 97 patients (45.54%). According to the Kelly classification, leaks were graded as follows: Grade 1 in 41 patients (19.25%), Grade 2 in 45 patients (21.13%), and Grade 3 in 11 patients (5.16%). The mean operative duration was 117.3 ± 42.5 min (Table 2).

**Table 2:** Intraoperative and postoperative outcomes (*n* = 213).

Outcome	Sum
Intraoperative CSF leak (Kelly grade)	
0	116 (54.46%)
1	41 (19.25%)
2	45 (21.13%)
3	11 (5.16%)
Postoperative complications	
CSF rhinorrhea	3 (1.41%)
Meningitis	4 (1.88%)
Hyposmia (3-month)	2 (0.94%)

### 3.3 Postoperative complications

**Figure 3:**
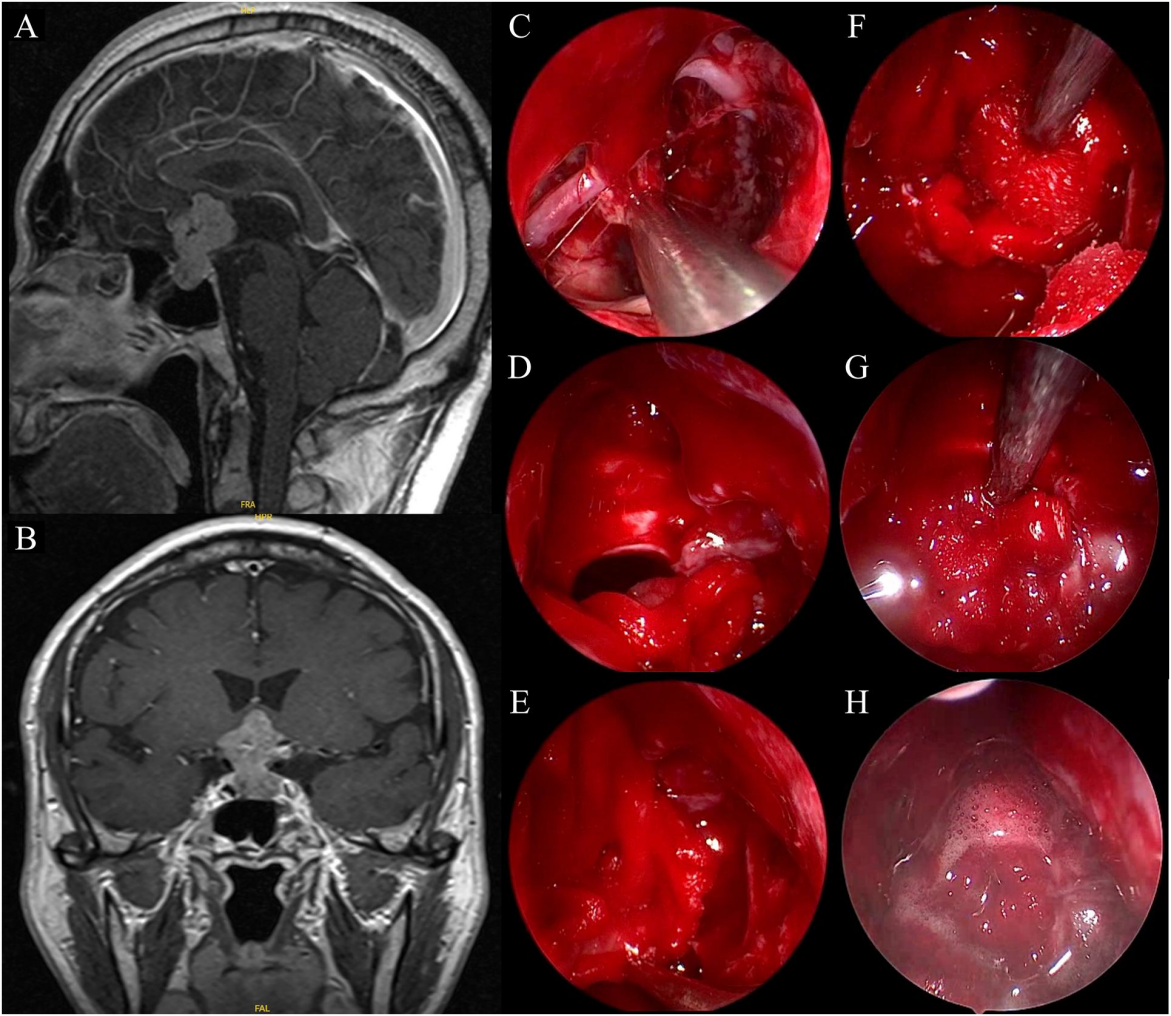
A typical case of grade 3 intraoperative leakage. Male, 54 years old, admitted for decreased vision in both eyes. **(A,B)** Preoperative MR demonstrates a pituitary tumor with suprasellar extension and inferior invasion into the sphenoid sinus. **(C)** A massive defect in the diaphragma sellae reveals clearly visible vessels within the suprasellar cistern. **(D,E)** Cover the entire sellar region with artificial dura mater. **(F,G)** Fill the tumor cavity with gelation sponge to ensure tightly adhesion between artificial dura mater and sellar diaphragma. **(H)** Sealing with porcine fibrin sealant.

Within three months postoperatively, CSF rhinorrhea developed in three patients (1.41%; 95% CI: 0.29%–4.25%). Among the 97 patients with intraoperative CSF leakage, the postoperative CSF rhinorrhea rate was 3.1% (3/97; 95% CI: 0.67%–9.08%). A descriptive comparison of baseline characteristics between patients with and without postoperative CSF rhinorrhea is presented in Table 3. The clinical details of the three affected patients are summarized in Table 4. Of these three patients, one had an intraoperative Grade 1 leak, and two had a Grade 2 leak. None required surgical revision. The patient with CSF rhinorrhea alone was treated with LD for 5–7 days combined with bed rest and head elevation. The other two patients developed concurrent meningitis and received intravenous antibiotics (meropenem plus vancomycin) in addition to LD. All three leaks resolved without repeat endonasal surgery. Among the 11 Grade 3 patients, no postoperative leak was observed. Figure 3 shows a representative case of a Grade 3 intraoperative leak that did not develop postoperative CSF rhinorrhea after receiving the triple-layer reconstruction and standardized postoperative management (Table 2).

**Table 3:** Descriptive comparison of patients with and without postoperative CSF leak.

Characteristic	No postoperative leak (*n* = 210)	Postoperative (*n* = 3)
Age (mean ± SD)	52.63 ± 13.04	51.7 ± 10.50
Female	124 (59.05%)	2 (66.67%)
Macroadenoma/giant adenomas	183 (87.14%)	3 (100%)
Knosp grade 3-4	51 (24.29%)	2 (66.67%)
Intraoperative leak (any grade)	94 (44.76%)	3 (100%)
Grade 1	40 (19.05%)	1 (33.33%)
Grade 2	43 (20.48%)	2 (66.67%)
Grade 3	11 (5.24%)	0 (0%)

No inferential statistical testing was performed due to the small number of events (*n* = 3). Moreover, the number of Grade 3 intraoperative leaks (*n* = 11) is too small to support any conclusion regarding efficacy in high-flow defects. Data are presented descriptively only.

**Table 4:** Characteristics of 3 patients with postoperative CSF rhinorrhea.

Patient	Gender	Age	Pathology	Knosp grade	Maximal tumor diameter (mm)	Intraoperative CSF leak (Kelly grade)	Time of onset	Management	Meningitis	Hyposmia (3-month)
1	F	52	NF	4	28	II	Postoperative day 5 (in-hospital)	LD	No	No
2	F	62	NF	1	45	II	Postoperative day 2 (in-hospital)	LD + antibiotics	Yes	No
3	F	41	NF	3	25	I	Postoperative day 12 (after discharge)	LD + antibiotics	Yes	No

CSF, cerebrospinal fluid; LD, lumbar drainage.

Postoperative meningitis was diagnosed in four patients (1.88%). All four were culture-positive. Two had concurrent postoperative CSF rhinorrhea. The other two had no postoperative rhinorrhea but had intraoperative Grade 3 and Grade 2 leaks. All four received LD plus intravenous antibiotics (meropenem plus vancomycin) and recovered without sequelae.

Due to the small number of events (*n* = 3 for CSF rhinorrhea), formal statistical comparison among Kelly grades was not performed; the data are presented descriptively.

### 3.4 Olfactory function outcomes

At the 3-month postoperative follow-up, two patients (0.94%) reported subjective hyposmia. Among the 197 patients who completed the 6-month follow-up, both affected patients regained normal olfactory function (Table 2).

## 4 Discussion

### 4.1 Principal findings and clinical context

Postoperative CSF rhinorrhea continues to be the most common complication following EEA for resection of sellar lesions, and its management plays a decisive role in determining patient outcomes. In this retrospective series of 213 patients undergoing standard EEA for pituitary adenomas, a simple triple-layer reconstruction strategy—utilizing artificial dura, gelatin sponge, and porcine fibrin sealant— was associated with a postoperative CSF leak rate of 1.41% (3/213) overall. Among the 97 patients with intraoperative CSF leakage, the postoperative leak rate was 3.1% (3/97). For context, multicenter studies using more complex, multi-layer repairs have reported postoperative leak rates ranging from 2.0% to 6.0% (3, 17, 19, 21, 23–25) Direct cross-study comparisons are not valid due to differences in patient selection, leak grading, and outcome definitions. Regarding the 11 patients with Kelly Grade 3 intraoperative leaks, no postoperative CSF rhinorrhea was observed in this series; however, given the small sample size, this finding should be interpreted with caution. In addition, the technique eliminated donor-site morbidity and was associated with a very low rate of transient olfactory disturbance (0.94%), which resolved spontaneously in all cases by six months.

### 4.2 Rationale and comparative effectiveness

**Figure 4:**
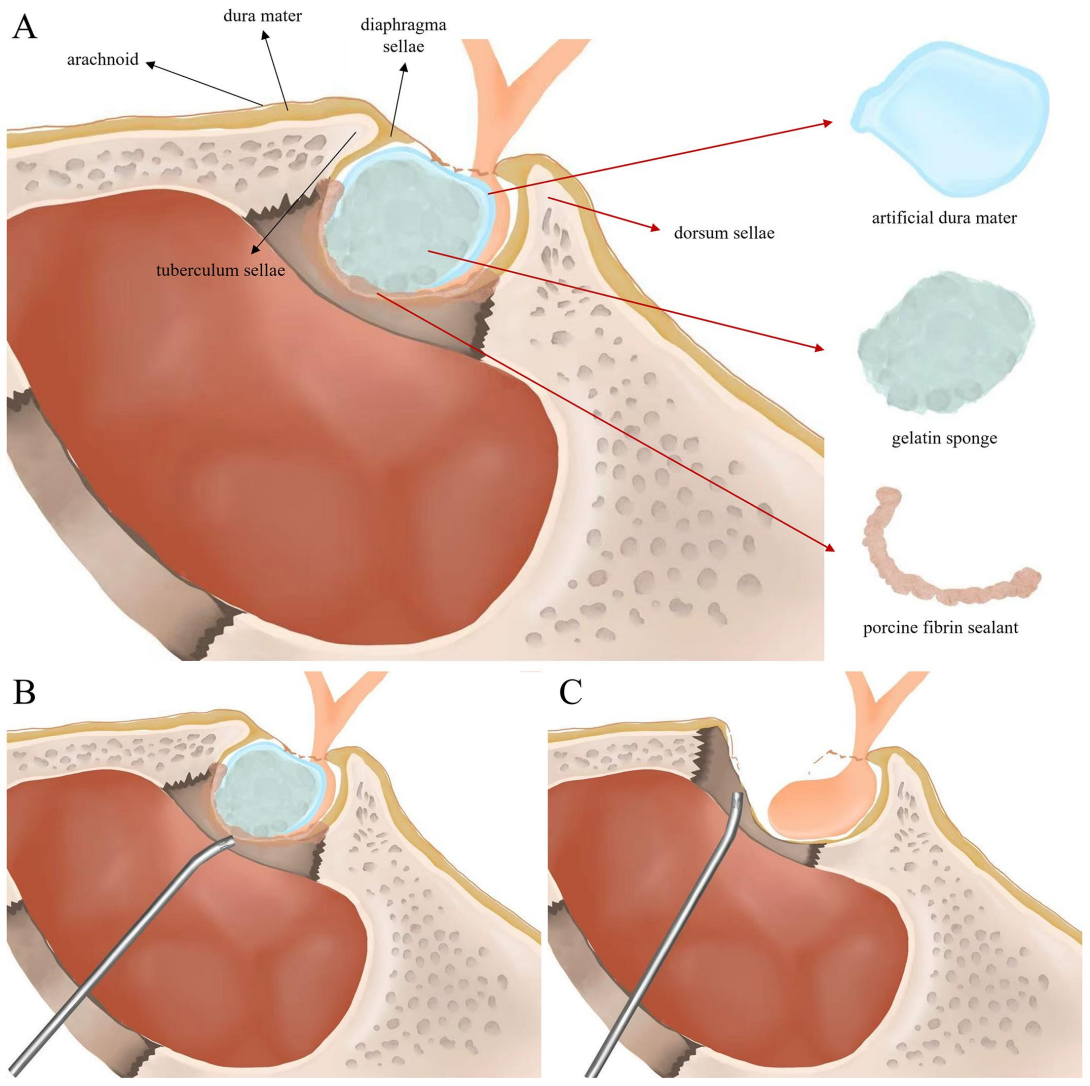
Schematic diagram. **(A)** Schematic illustration of the triple-layer reconstruction strategy. **(B)** Standard EEA, showing CSF leakage originating from the arachnoid membrane of the diaphragma sellae. **(C)** Expanded EEA (trans-tuberculum approach), showing a large suprasellar dural defect where the simple triple-layer reconstruction would be insufficient.

The efficacy of this simplified approach can be attributed to its alignment with the anatomical reality of standard EEA. Unlike expanded EEA procedures (26) where large dural defects are created, CSF leaks in standard EEA predominantly originate from the arachnoid layer of sellar diaphragm (3, 9, 27). Our technique addresses this specifically: the artificial dura provides a direct barrier; the gelatin sponge generates uniform, sustained pressure to appose the arachnoid; and the biological gel consolidates the seal. The preserved corners of the cruciate dural incision offer external support (Figure 4). This targeted, multi-point stabilization facilitates the arachnoid's innate healing capacity without requiring the technical complexity and donor-site cost of a VNSF (28–31) While VNSF remains valuable for extensive defects, the present results indicate that a simpler, less invasive reconstruction achieved leak rates within the range reported for more complex techniques in this case series of standard EEA cases for primary pituitary adenomas. Our postoperative leak rate of 1.41% is within the range of those reported for more elaborate techniques (Table 5). However, as shown in Table 5, direct comparisons across studies are limited by significant heterogeneity in patient populations, reconstruction algorithms, intraoperative leak grading systems, and outcome definitions. Therefore, these comparisons are descriptive only and should not be interpreted as evidence of equivalence or superiority of any single technique. In this series, self-reported hyposmia was noted in 2 patients (0.94%), both of whom recovered normal olfaction by 6 months. However, because olfactory function was assessed only by patient self-report, the true incidence may be higher. In comparison, studies of VNSF techniques have reported higher rates of smell disturbance (29, 30). All told, this simplified strategy helps streamline the operation, lowers the risk of nasal complications, and avoids donor-site issues such as pain and infection.

**Table 5:** Descriptive comparison of reconstruction outcomes with other series.

Study	Study type	Sample Size	Tumor type	Surgical approach	Intraop leak grading	Reconstruction strategy (by grade)	Overall postop CSF leak rate	Postop leak rate in intraop leak group	Meningitis rate	Prophylactic LD use
Baussart et al. (33)	Retrospective, single-center	3015	Pituitary adenoma	Standard	Modified Esposito	Oozing: TachoSil	1%	3.4% (11/319)	0.8%	Abandoned (replaced by Foley balloon after 2014)
						Low-flow: Epidural graft (middle turbinate/fascia lata)				
						High-flow: Intrasellar fascia + muscle				
						High-risk (BMI > 40, redo, prior RT): Foley balloon or double NSF				
Zhang et al. (34)	Retrospective, single-center	703	Pituitary adenoma	Standard	Esposito-Kelly grade 0-3	G1: Fat packing + artificial dura	0.14%	1.16% (1/86, only in G2)	Not reported	None
						G2: “Bathtub plug” fat + artificial dura				
						G3: Autologous fascia continuous suturing + NSF				
Shen et al. (32)	Retrospective with PSM	459 (all G1)	Pituitary adenoma (92.4%), Rathke's cyst, craniopharyngioma	Standard	Only grade 1	Group A: Collagen sponge (“baseball cap” technique)	2.4% (in G1 patients)	Group A: 1.0%, Group B: 1.4%, Group C: 35.3%	Not reported	Excluded (patients with LD were excluded)
						Group B: Autologous fat graft				
						Group C: Gel foam + bipolar coagulation only				
Zeden et al. (15)	Retrospective, single-center	30	Pituitary macroadenoma (25), Rathke's cyst (5)	Standard	Custom 4-tier (-, +, ++, +++)	Sellar filling (grade-dependent): GS/CM/ OC/abdominal fat	3.3% (1/30)	leak occurred in patient with no intraop leak	0%	None
						All grades: Rigid epidural PDS foil + fibrin glue				
Fishpool et al. (35)	Prospective, single-center	32	Pituitary adenoma	Standard	Not formally graded	All grades: Free middle turbinate mucosal graft + Tisseel (fibrin glue) + Gelfoam packing (no rigid buttress, no nasal packing, no LD)	0% (0/32)	3 intraop leaks, all repaired successfully	Not reported	None
Scagnelli et al. (36)	Retrospective, single-center	122	Pituitary adenoma	Standard	Not formally graded (low vs. high flow)	All low-grade leaks: Nasal floor free mucosal graft (overlay) + Durepair (inlay)	0.82% (1/122)	Not reported (39% intraop leak rate)	Not reported	None
						High-flow (cistern opened): NSF (9 cases, excluded from this group)				
This study	Retrospective, single-center	213	Pituitary adenoma	Standard	Esposito-Kelly grade 0-3	Triple-layer (all grades, no autograft, no NSF): Artificial dura (overlay) + gelatin sponge packing + biological protein gel sealing	1.41%	G1: 2.4% (1/41), G2: 4.4% (2/45), G3: 0% (0/11)	1.88%	None

Direct comparisons across studies are limited by differences in patient populations, surgical approaches, reconstruction protocols, and outcome definitions. The data are presented for descriptive contextual purposes only. No conclusions regarding equivalence or superiority can be drawn from this table. CSF, cerebrospinal fluid; LD, lumbar drainage; TachoSil, collagen sponge coated with human coagulation factors (fibrinogen/thrombin); BMI, body mass index; RT, radiotherapy; NSF, nasoseptal flap (vascularized); PSM, propensity score matching; GS, gelatin sponge; CM, collagen matrix; OC, oxidized cellulose; PDS, polydioxanone.; G (grade), Esposito-Kelly leak grade (0 = no leak, 1 = weeping, 2 = moderate, 3 = high-flow).

A study from Huashan Hospital (32) used a horseshoe-shaped dural incision during EEA. After tumor removal, they placed DuraGen over the anterior sellar diaphragm, filled the cavity with gelatin sponge, and reinforced it externally with Ethisorb—a method that worked well for Grade 1 leaks. Although the horseshoe incision may improve exposure for some lesions, standard EEA generally targets intrasellar pathology, and with modern endoscopic visualization, the cruciate incision offers comparable exposure while preserving more natural support. In our series, no postoperative CSF rhinorrhea was observed among the 11 patients with Grade 3 intraoperative leaks. However, given the small sample size, this finding is preliminary and requires confirmation in larger studies.

### 4.3 Insights from failures and limitations

In our series, three patients (1.41%) developed postoperative CSF rhinorrhea. A close review of these cases revealed that each had experienced abrupt rises in intracranial pressure—due to constipation, coughing, or sneezing—before the leak became apparent. This likely disrupted the healing interface before it had fully stabilized. All three patients were successfully managed with LD and conservative measures, without the need for surgical revision. These instances suggest that our reconstruction remains effective under normal, resting conditions, and highlight that patient counseling—especially about avoiding Valsalva maneuvers—is as important as the surgical technique itself. It is also noteworthy that these three patients had higher Knosp grades and larger tumors, suggesting that larger resection cavities and possibly thinner, irregular sellar diaphragms may add to the complexity of reconstruction. Such cases call for especially careful postoperative management and patient guidance. Regarding the four meningitis cases, all were culture-positive and received LD plus intravenous antibiotics. Two of these patients had concurrent postoperative CSF rhinorrhea (described above). The other two had no postoperative rhinorrhea but had intraoperative Grade 3 and Grade 2 leaks. These two cases without postoperative CSF rhinorrhea were attributed to retrograde bacterial infection through the intraoperative CSF leak site, although alternative mechanisms cannot be excluded, including intraoperative bacterial contamination during surgery, hematogenous spread from a distant source, or suboptimal coverage by prophylactic antibiotics. These two cases highlight that intraoperative CSF leakage is a risk factor for meningitis independent of postoperative rhinorrhea. Clinical suspicion for meningitis should remain high in patients with intraoperative leaks, even without nasal discharge, and prompt CSF evaluation is warranted when suggestive symptoms occur. Optimized perioperative antibiotic strategies and rigorous aseptic technique are also important for reducing this complication.

This study has several limitations. Its retrospective, single-center design and lack of a control group preclude definitive causal conclusions. Olfactory assessment relied on patient self-report rather than validated testing, so our 0.94% hyposmia rate is likely a lower-bound estimate. The small number of Grade 3 leaks (*n* = 11) limits conclusions for high-flow cases. The 3-month follow-up, while adequate for capturing most CSF leaks, is short for long-term outcomes. We focused only on reconstruction-related outcomes and did not assess resection extent, endocrine remission, or visual recovery. Our findings apply only to primary pituitary adenomas via standard EEA, not to expanded approaches, recurrent tumors, or prior radiotherapy. Finally, variations in patient compliance with postoperative protocols may have influenced outcomes.

## 5 Conclusions

In conclusion, in this retrospective, descriptive, consecutive case series, the triple-layer reconstruction strategy was associated with a postoperative CSF leak rate of 3.1% among patients with intraoperative CSF leakage following standard EEA for primary pituitary adenomas. This technique avoided autograft harvest and donor-site morbidity. However, this study reports only reconstruction-related outcomes; other important surgical outcomes—including gross total resection, endocrine remission, visual recovery, and diabetes insipidus—were not assessed. Due to the absence of a control group and the small number of Grade 3 leaks (*n* = 11), these findings should be considered preliminary, and prospective controlled studies are needed.

## Data Availability

The original contributions presented in the study are included in the article/Supplementary Material, further inquiries can be directed to the corresponding authors.

## References

[1] Jho HD Carrau RL. Endoscopic endonasal transsphenoidal surgery: experience with 50 patients. J Neurosurg. (1997) 87(1):44–51. 10.3171/jns.1997.87.1.0044 9202264

[2] Prevedello DM Doglietto F Jane JA Jagannathan J Han J Laws ER. History of endoscopic skull base surgery: its evolution and current reality. J Neurosurg. (2007) 107(1):206–13. 10.3171/JNS-07/07/0206 17639897

[3] Khan DZ Ali AMS Koh CH Dorward NL Grieve J Layard Horsfall H. Skull base repair following endonasal pituitary and skull base tumour resection: a systematic review. Pituitary. (2021) 24(5):698–713. 10.1007/s11102-021-01145-4 33973152 PMC8416859

[4] Sciarretta V Mazzatenta D Ciarpaglini R Pasquini E Farneti G Frank G. Surgical repair of persisting CSF leaks following standard or extended endoscopic transsphenoidal surgery for pituitary tumor. Minim Invasive Neurosurg. (2010) 53(2):55–9. 10.1055/s-0029-1246161 20533135

[5] Wang EW Zanation AM Gardner PA Schwartz TH Eloy JA Adappa ND. ICAR: endoscopic skull-base surgery. Int Forum Allergy Rhinol. (2019) 9(S3):S145–365. 10.1002/alr.22326 31329374

[6] Zhang C Yang N Mu L Wu C Li C Li W. The application of nasoseptal “rescue” flap technique in endoscopic transsphenoidal pituitary adenoma resection. Neurosurg Rev. (2020) 43(1):259–63. 10.1007/s10143-018-1048-8 30535967 PMC7010618

[7] Zhang C Ding X Lu Y Hu L Hu G. Cerebrospinal fluid rhinorrhoea following transsphenoidal surgery for pituitary adenoma: experience in a Chinese centre. Acta Otorhinolaryngol Ital. (2017) 37(4):303–7. 10.14639/0392-100X-1086 28872159 PMC5584102

[8] Martin C Leclercq D Boch AL Jublanc C Kuhn E. Determinants of cerebrospinal fluid leakage in a large cohort of macroprolactinomas. [2213-3941 (Electronic)]. 10.1016/j.ando.2025.101685 39818291

[9] Kim EH Roh TH Park HH Moon JH Hong JB Kim SH. Direct suture technique of normal gland edge on the incised dura margin to repair the intraoperative cerebrospinal fluid leakage from the arachnoid recess during transsphenoidal pituitary tumor surgery. Neurosurgery. (2015) 11(Suppl 2):26–31. 10.1227/NEU.0000000000000612 discussion. 25584954

[10] Lasica N Lesha E Beckfort NS Arnautovic KI. Does the crafted abdominal fat grafting technique completely eliminate risk of postoperative CSF leak in endonasal pituitary surgery? Technical note and preliminary clinical outcome. Neurosurg Focus. (2025) 58(2):E3. 10.3171/2024.11.FOCUS24665 39891940

[11] Erkan B Demir S Akpinar E Hasimoglu O Baskan F Cirak M. Effectiveness of a sellar reconstruction algorithm in transsphenoidal pituitary surgery: insights from 490 cases. World Neurosurg. (2024) 189:e1098–e108. 10.1016/j.wneu.2024.07.093 39032635

[12] Zhao WJ Yang G Li RC Huo G Gao D Cao MC. Effects of cruciate embedding fascia-bone flap technique on grade II-III cerebral spinal fluid leak in endoscopic endonasal surgery. BMC Surg. (2022) 22(1):288. 10.1186/s12893-022-01730-9 35883063 PMC9327233

[13] Soudry E Turner JH Nayak JV Hwang PH. Endoscopic reconstruction of surgically created skull base defects: a systematic review. Otolaryngol Head Neck Surg. (2014) 150(5):730–8. 10.1177/0194599814520685 24493791

[14] Leng LZ Brown S Anand VK Schwartz TH. “Gasket-seal” watertight closure in minimal-access endoscopic cranial base surgery. Neurosurgery. (2008) 62(5 Suppl 2):ONSE342–3. 10.1227/01.neu.0000326017.84315.1f 18596534

[15] Zeden JP Baldauf J Schroeder HWS. Repair of the sellar floor using bioresorbable polydioxanone foils after endoscopic endonasal pituitary surgery. Neurosurg Focus. (2020) 48(6):E16. 10.3171/2020.3.FOCUS2064 32480371

[16] Nix P Tyagi A Phillips N. Retrospective analysis of anterior skull base CSF leaks and endoscopic repairs at Leeds. Br J Neurosurg. (2016) 30(4):422–6. 10.3109/02688697.2016.1161176 27008345

[17] Jin J Shen H Peng G Zhou J Xu D Chen Y. The role of comprehensive structural preservation strategy in skull base reconstruction following endoscopic endonasal surgery for pituitary neuroendocrine tumors: a retrospective single-center study. World Neurosurg. (2025) 200:124171. 10.1016/j.wneu.2025.124171 40505855

[18] Moon JH Kim EH Kim SH. Snare technique for the remodeling of the redundant arachnoid pouch to prevent cerebrospinal fluid rhinorrhea and hematoma collection during transsphenoidal surgery for suprasellar-extended pituitary tumors. J Neurosurg. (2016) 125(6):1443–50. 10.3171/2015.11.JNS151328 26967785

[19] Moon JH Kim EH Kim SH. Various modifications of a vascularized nasoseptal flap for repair of extensive skull base dural defects. J Neurosurg. (2020) 132(2):371–9. 10.3171/2018.10.JNS181556 30738381

[20] Knosp E Steiner E Kitz K Matula C. Pituitary adenomas with invasion of the cavernous sinus space: a magnetic resonance imaging classification compared with surgical findings. Neurosurgery. (1993) 33(4):610–7. 10.1097/00006123-199310000-00008 discussion 7–8. 8232800

[21] Esposito F Dusick JR Fatemi N Kelly DF. Graded repair of cranial base defects and cerebrospinal fluid leaks in transsphenoidal surgery. Oper Neurosurg. (2007) 60(4 Suppl 2):295–303. 10.1227/01.NEU.0000255354.64077.66 discussion –4. 17415166

[22] Cong Z Zhu J Sun H Tang C Yang J Ma C. Endoscopic 1½-transseptal approach for pituitary surgery. Front Oncol. (2022) 12:1116408. 10.3389/fonc.2022.1116408 36713529 PMC9877324

[23] Ciric I Ragin A Baumgartner C Pierce D. Complications of transsphenoidal surgery: results of a national survey, review of the literature, and personal experience. Neurosurgery. (1997) 40(2):225–36. 10.1097/00006123-199702000-00001 discussion 36–7. 9007854

[24] Cai X Yang J Zhu J Tang C Cong Z Liu Y. Reconstruction strategies for intraoperative CSF leak in endoscopic endonasal skull base surgery: systematic review and meta-analysis. Br J Neurosurg. (2022) 36(4):436–46. 10.1080/02688697.2020.1849548 33475004

[25] Cappabianca P Cavallo LM Colao A De Divitiis E. Surgical complications associated with the endoscopic endonasal transsphenoidal approach for pituitary adenomas. J Neurosurg. (2002) 97(2):293–8. 10.3171/jns.2002.97.2.0293 12186456

[26] Kassam AB Snyderman C Gardner P Carrau R Spiro R. The expanded endonasal approach: a fully endoscopic transnasal approach and resection of the odontoid process: technical case report. Neurosurgery. (2005) 57(1 Suppl):E213. 10.1227/01.NEU.0000163687.64774.E4 discussion E. 15987596

[27] Roca E Penn DL Safain MG Burke WT Castlen JP Laws ER. Abdominal fat graft for sellar reconstruction: retrospective outcomes review and technical note. Oper Neurosurg. (2019) 16(6):667–74. 10.1093/ons/opy219 30124966

[28] Sonnenburg RE White D Ewend MG Senior B. Sellar reconstruction: is it necessary? Am J Rhinol. (2003) 17(6):343–6. 10.1177/194589240301700605 14750609

[29] Charalampaki P Ayyad A Kockro RA Perneczky A. Surgical complications after endoscopic transsphenoidal pituitary surgery. J Clin Neurosci. (2009) 16(6):786–9. 10.1016/j.jocn.2008.09.002 19289287

[30] Zhu J Feng K Tang C Yang J Cai X Zhong C. Olfactory outcomes after endonasal skull base surgery: a systematic review. Neurosurg Rev. (2021) 44(4):1805–14. 10.1007/s10143-020-01385-1 32914235

[31] Cardinal T Brunswick A Strickland BA Micko A Shiroishi M Liu C-SJ. Safety and effectiveness of the direct endoscopic endonasal approach for primary sellar pathology: a contemporary case series of more than 400 patients. World Neurosurg. (2021) 148:e536–46. 10.1016/j.wneu.2021.01.018 33454431

[32] Shen M Qiao N Shou X Chen Z He W Ma Z. Collagen sponge is as effective as autologous fat for grade 1 intraoperative cerebral spinal fluid leakage repair during transsphenoidal surgery. Clin Neurol Neurosurg. (2022) 214:107131. 10.1016/j.clineuro.2022.107131 35134707

[33] Baussart B Venier A Jouinot A Reuter G Gaillard S. Closure strategy for endoscopic pituitary surgery: experience from 3015 patients. Front Oncol. (2022) 12:1067312. 10.3389/fonc.2022.1067312 36686774 PMC9846073

[34] Zhang C Yang Z Liu P. Strategy of skull base reconstruction after endoscopic transnasal pituitary adenoma resection. Front Surg. (2023) 10:1130660. 10.3389/fsurg.2023.1130660 36998598 PMC10043245

[35] Fishpool SJ Amato-Watkins A Hayhurst C. Free middle turbinate mucosal graft reconstruction after primary endoscopic endonasal pituitary surgery. Eur Arch Otorhinolaryngol. (2017) 274(2):837–44. 10.1007/s00405-016-4287-8 27586390

[36] Scagnelli RJ Patel V Peris-Celda M Kenning TJ Pinheiro-Neto CD. Implementation of free mucosal graft technique for sellar reconstruction after pituitary surgery: outcomes of 158 consecutive patients. World Neurosurg. (2019) 122:e506–11. 10.1016/j.wneu.2018.10.090 30368014

